# Tapping-lips aggravated interictal bilateral discharges in EEG in the patients with Rett syndrome: a case report

**DOI:** 10.1186/s12883-019-1296-4

**Published:** 2019-05-01

**Authors:** Xiaoqing Luo, Cheng Li, Xiaolu Wang, Xiaoli Yu, Jun Jiang

**Affiliations:** Department of electrophysiology, Wuhan Children’s Hospital (Wuhan Maternal and Child Healthcare Hospital), Tongji Medical college, Huazhong University of Science&Technology, Wuhan, Hubei China

**Keywords:** Rett syndrome, Electroencephalogram, Reflex discharges, Precipitated by tapping- lips

## Abstract

**Background:**

Rett syndrome (RTT) is a severe neurodevelopmental disorder mainly affecting females. One of the main clinical manifestations is hand stereotypies, which is presumed to based on dysfunction rather than on structural impairment. Reflex interictal discharge precipitated by tapping-lips in electroencephalogram (EEG) is a rare phenomenon among RTT.

**Case presentation:**

We firstly reported a case of RTT concerning interictal reflex discharge precipitated by tapping- lips. The child, female, 5 years old, presented with a significant regression in motor development and language skills. She almost always tapped the lips with the right hand and stopped only when was interrupted. Her EEG results displayed extensive low amplitude fast wave could be elicited by lightly and slowly- rhythm tapping lips and multifocal bilateral discharges could be precipitated by relatively stronger and quicker rhythm action. It was when the movement stopped that corresponding discharges immediately disappeared. Besides, the reflex discharges were not precipitated by tapping- lips using observer’s hand at the certain tempo and intensity. The hand stereotypies did not respond to antiepileptic drugs.

**Conclusions:**

Tapping- lips may be a somatosensory stimulation to precipitate interictal discharges in RTT, which may provide another idea to enrich the insight on hand stereotypies of RTT.

## Background

Rett syndrome (RTT) is a neurodevelopmental disorder predominantly affecting females and characterized by mental retardation, stereotypical hand movements, and seizure [1, 2]. It is primarily caused by mutations in the methyl-CpG-binding protein 2 (MECP2) gene on Xq28, which encodes MECP2 [3, 4]. Classical RTT is diagnosed based on a set of clinical criteria. Epilepsy is reported to occur in 50 to 90% of individuals with Rett syndrome, characterized by complex partial, atypical absence and generalized tonic–clonic [5, 6]. The electroencephalogram (EEG) abnormalities in RTT are well documented, mainly a slowing of the background activity and bilateral central spikes aggravating during sleep [5, 6]. Reflex interictal discharge is a rare phenomenon among RTT, primarily triggered by various kinds of visual stimuli. We reported one child diagnosed with RTT with partial seizure and a specific EEG feature that rhythmic tapping- lips triggered and aggravated multifocal bilateral discharges in EEG. To our knowledge, this report is the first to present tapping- lips may be a precipitating factor of reflective discharges in RTT, which may provide a new clue to understand the mechanisms of some symptoms of RTT.

## Case presentation

**Fig. 1:**
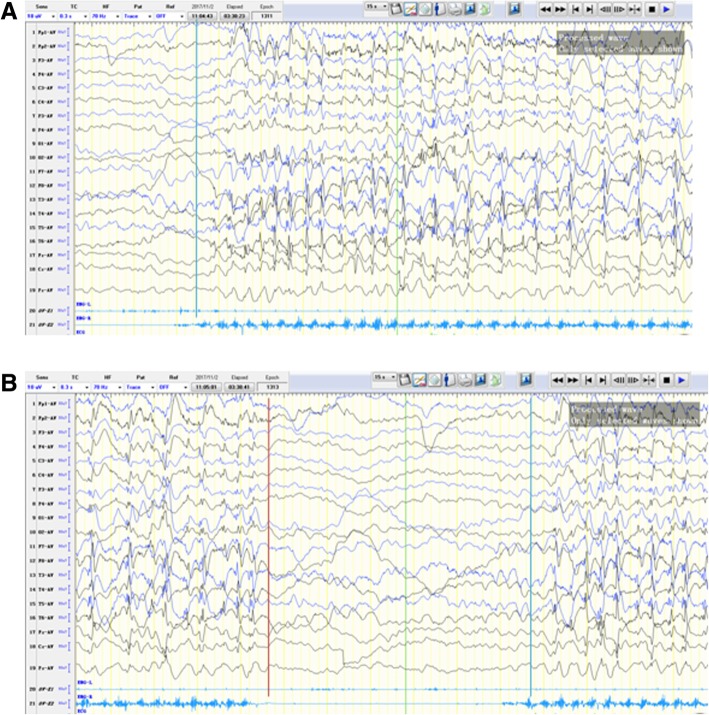
**a** and **b** represent the beginning and end of an action and the beginning of the next action. EEG results displayed stronger and faster rhythm action precipitated multifocal bilateral discharges. EMG on both deltoid muscles recorded the action. The green line represents the beginning of tapping-lips, and red line represents the end of tapping-lips

**Fig. 2:**
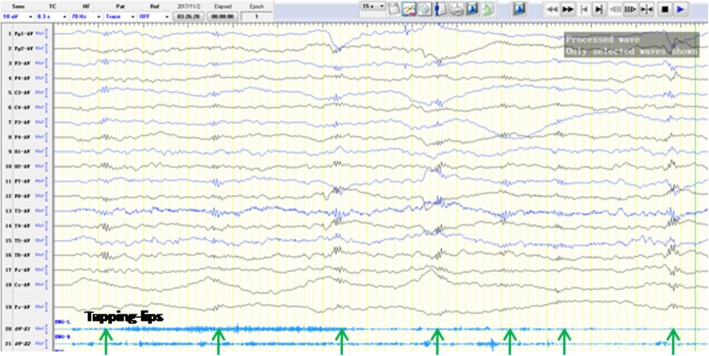
EEG results displayed extensive low amplitude fast wave rhythm could be elicited by lightly and slowly- rhythm tapping lips. EMG on both deltoid muscles recorded the action

The case was a 5-year-old-girl, who was admitted to the hospital with a 2-year history of intermittent convulsions. She was born to healthy, unrelated parents. Her mother had slight vaginal bleeding for one week at the three months pregnancy without any therapy. She appeared normal at birth at 40 + 1 weeks of gestation and had normal Apgar scores. Birth weight, length, and head circumference were within normal ranges. Family history was unremarkable for neurologic diseases. Her psychomotor development was delayed. Her parents reported that she sat at eight months, and walked with support until 18 months. She suffered from bruxism and characteristic stereotyped hand movements such as clapping and tapping started at the age of 2 years. At the age of 3 years, she developed seizures characterized by staring eyes, hands rubbing and hypokinesis of the head (10 times per day), severely associated with lip and face cyanosis (1 time per day). Her parents reported that her EEG in a specific hospital showed abnormality at three years of age, and the seizures were not adequately controlled after one year of Chinese medicine therapy. Thus, they gave up drug therapy. In the last year, another abnormal behavior was observed. She regularly tapped her lips using the right hand, and bed using the left hand in awake and stopped until sleep. In the last month, the frequency of seizure increased significantly, and seizure symptoms developed more serious. So her parents took her to our hospital for comprehensive examination. She was diagnosed with RTT from her history and clinical findings. A cerebral magnetic resonance image was normal. Mutational analysis of MECP2 revealed a mutation in this affected girl. Twenty-four-hour video-EEG recordings revealed a slowing advantage rhythm (5-6 Hz) of occipital region and extensive slow wave activity in the background activity. Besides, multifocal discharges were noted during awake, especially in the Rolandic region, significantly aggravated to evolution toward electrical status epilepticus during sleep (ESES). Several partial seizures and constantly-tapping her lips using right hand were recorded during EEG recordings, which was the same as that described by the parents. To our surprise, tapping- lips in light and slow rhythm could trigger extensive low amplitude fast wave rhythm (Fig. 1), and relatively stronger and quicker rhythm could result in multifocal bilateral discharges (Fig. 2). Corresponding discharges were disappeared when the movement was intervened by parents. The patient chose left hand to rhythmically tap lips when the right hand was restricted, but there were no synchronic discharges accompanied by the action. Moreover, the synchronous phenomenon of action and discharge was not observed when the right hand was tapping cheek or abdomen. More interestingly, the discharges were not precipitated by tapping- lips using observer’s hand at the certain tempo and intensity. The coexistence phenomenon persisted in different EEG caps, instruments, and rooms, which ruled out the possibility of an artifact. By the phone call following-up, the total frequency of epilepsy seizures was markedly less after one year of treatment with valproate, but hand stereotypies did not respond to the antiepileptic drug.

## Discussion and conclusions

We presented a unique case of RTT that rhythmic tapping- lips triggered or aggravated interictal multifocal bilateral discharges in EEG. Several spontaneous partial seizures were recorded during the 24 h-EEG recording. EEG results displayed extensive low amplitude fast wave rhythm could be elicited by lightly and slowly- rhythm tapping lips and multifocal bilateral discharges could be precipitated by relatively stronger and quicker rhythm action. It was when the action stopped that corresponding discharges immediately disappeared. There were no evident clinical manifestations of epileptic seizures in the process of rhythmic tapping-lips. Moreover, the coexistence phenomenon of action and discharge was not observed when the right hand tapping cheek or abdomen, the left hand tapping lips, or observer’s hand tapping the lips. It was an uncommon and unreported the feature for Rett syndrome.

Epileptic seizure stems from abnormal electrical activity in the brain, causing involuntary movement or behavior, in other words, abnormal electrical activity precedes the clinical seizures [7]. It is widely accepted that epileptic seizure and ictal EEG were not immediately interrupted by external factor. Our patient showed that motor activity preceded electrographic discharges from the point of electrical activity of the deltoid muscle in Fig. 1, and was passively terminated at once. The electrographic discharges immediately disappeared with the motor activity. Moreover, hand stereotypies of tapping-lips did not respond to the antiepileptic drug. These characteristics make the possibility of epilepsy unlikely, and reflex epilepsy is no exception. It was reported that stereotypic hand movements should not be accompanied by any specific EEG discharges [8]. In our case, lightly and slowly- rhythm tapping lips elicited extensive low amplitude fast wave rhythm, and relatively stronger and faster rhythm action precipitated multifocal bilateral discharges. The patient proactively chose left hand to rhythmically tap lips when right hand was restricted, although there were no synchronic discharges accompanied by the action. The above phenomenon supports that tapping-lips is not a simple hand stereotype, possibly making the patient feel some kind of mental comfort.

There have been very few reports on the relationship between stereotyped hand movements and discharges in RTT. Lv et al. reported that a girl with RTT and unique EEG findings consisting of continuous centrotemporal spikes, which can be suppressed by the repeated hand clapping [9]. Niedermeyer and Naida provided four cases of the Rett syndrome, where paroxysmal activity and especially spike discharges over the central region could be blocked or attenuated by passive finger movements [10]. The two reports showed that stereotyped hand movements had an inhibitory effect on discharges. Nissenkorn A and Ben-Zeev B described a peculiar type of unilateral, highly rhythmic hand tapping accompanied by contralateral synchronous centrotemporal spikes on electroencephalography in patients with RTT [11]. Our case presented that interictal multifocal bilateral discharges could be precipitated by tapping- lips. The two reports presented that stereotyped hand movements may trigger discharges. These reports of RTT further described that stereotyped hand movements were not just simple action, occasionally could block or precipitate the interictal discharges. There may be one similar pathway between stereotyped hand movements in spite of the opposite effect in RTT. Nissenkorn A and Ben-Zeev B described cases of contralateral synchronous discharge, while our patient presented bilateral discharges, implying that brain networks may be involved in the mechanisms underlying reflex interictal discharge in RTT.

EEG recording of the case revealed that different frequency and intensity of tapping- lips led to different discharge forms. Lightly and slowly- rhythm tapping lips elicited extensive low amplitude fast wave rhythm, and relatively stronger and faster rhythm action precipitated multifocal bilateral discharges. Once the hand stereotypies stopped, the corresponding discharges immediately disappeared. The characteristic resembled that different intermittent photic stimulation can induce four types of EEG photoparoxysmal responses (PPR) [12, 13]: (I) spikes within the occipital rhythm, limited to the occipital regions (II) parieto-occipital spikes with a biphasic slow wave, (III) parieto-occipital spikes with a biphasic slow wave and spread to the frontal region, and (IV) generalised spikes and wave or polyspikes and wave. Thus, we hold the opinion that the phenomenon may be reflective discharge and that tapping- lips may be a new precipitating factor of reflective discharges in RTT. Types (I) and (II) of PPR are seen as unrelated to epilepsy. Type (III) and (IV) of PPR are considered abnormal. Especially type (IV) of PPR appears to be correlated with epilepsy. It was still unclear whether the reflective discharge precipitated by somatosensory stimulation was related to epilepsy, but there was a possibility. It was suggested that the mechanism underlying PPR may be relative to hyperexcitability of the primary visual cortex. The mechanism underlying discharges precipitated by tapping-lips may be implied with the hyperexcitable somatosensory cortex, in accord with the viewpoints stated by Nissenkorn A and Ben-Zeev B. In our case, reflex discharges were not precipitated by tapping- lips using observer’s hand at the certain tempo and intensity. The reason was that stimulus intensity or frequency were insufficient for triggering discharge, or that reflective discharge depended on the active motion of her own right hand. The coexistence phenomenon of action and discharge was not observed when the right hand tapping cheek or abdomen or the left hand tapping lips, implying that hyperexcitable somatosensory cortex may be specific in RTT. However, further studies will be required to the comprehensive understanding of mechanisms of reflex discharges precipitated by tapping- lips in RTT.

In conclusion, EEG recording revealed that different frequency and intensity of tapping- lips led to different discharge forms in RTT, implying that tapping- lips may be a somatosensory stimulation to precipitate interictal discharges in RTT.

## Abbreviations

EEG: electroencephalogram
ESES: electrical status epilepticus during sleep
MECP2: the methyl-CpG-binding protein 2
RTT: Rett syndrome
